# Parainfectious Transverse Myelitis in a Pregnant Woman: A Rare Clinical Entity

**DOI:** 10.7759/cureus.101420

**Published:** 2026-01-13

**Authors:** Akshatha Hegde, Akshatha V Rai, Anirudh Shetty

**Affiliations:** 1 Neurology, Manipal Hospital, Bengaluru, IND; 2 Critical Care Medicine, Justice KS Hegde Charitable Hospital, Mangaluru, IND; 3 Endocrinology, Diabetes and Metabolism, Narayana Health, Bengaluru, IND

**Keywords:** intravenous immunoglobulin, myelopathy, parainfectious, pregnancy, transverse myelitis

## Abstract

Transverse myelitis (TM) is an inflammatory disorder of the spinal cord characterized by motor, sensory, and autonomic impairments. Parainfectious transverse myelitis (PITM) represents a post-infectious, immune-mediated entity. Its occurrence during pregnancy is rare and presents with unique diagnostic and therapeutic challenges.

We report a 31-year-old primigravida at 32 weeks of gestation who presented with acute-onset bilateral lower limb weakness, sensory loss, and bladder involvement following a febrile illness. MRI spine revealed longitudinally extensive transverse myelitis (C3-T2). The cerebrospinal fluid analysis showed lymphocytic pleocytosis with a negative infectious and autoimmune work-up. She was treated with intravenous methylprednisolone, which was followed by intravenous immunoglobulin (2 g/kg over five days), with a gradual neurological recovery. She subsequently developed urinary sepsis at 35 weeks and underwent emergency cesarean section, delivering a preterm infant. At two months postpartum, she had near-complete motor recovery with only mild bladder urgency.

While PITM in pregnancy is rare, it is potentially reversible with timely immunomodulatory therapy. Intravenous immunoglobulin may be considered as a safe and effective adjunct to corticosteroids in selected cases.

## Introduction

Transverse myelitis (TM) is a rare immune-mediated inflammatory disorder of the spinal cord leading to varying degrees of motor, sensory, and autonomic impairment. The annual incidence is around one to eight cases per million population [1].

Parainfectious transverse myelitis (PITM) refers to postinfectious immune-mediated spinal cord inflammation, which is triggered by viral or bacterial pathogens [2]. It is frequently postinfectious or post-vaccination but may also arise in association with systemic autoimmune disorders (e.g., systemic lupus erythematosus or SLE) or primary autoimmune disorders of the central nervous system, such as neuromyelitis optica spectrum disorder (NMOSD), multiple sclerosis, and myelin oligodendrocyte glycoprotein antibody disease (MOGAD). Less commonly, transverse myelitis can result in paraneoplastic processes or exposure to certain drugs or toxins. Pathogenesis in NMOSD is primarily linked to the formation of antibodies against aquaporin 4 (AQP4) water channels, resulting in the clinical manifestations [3]. Pregnancy introduces additional diagnostic and therapeutic complexity due to its unique immunological milieu and the dual concern for maternal and fetal well-being. Reports of PITM during pregnancy are uncommon. We present a case of a pregnant woman who developed PITM in the third trimester and achieved near-complete recovery following treatment with intravenous corticosteroids and intravenous immunoglobulin.

## Case presentation

A 31-year-old previously healthy primigravida at 32 weeks of gestation presented with rapidly progressive bilateral lower limb weakness and numbness of two days' duration following a febrile illness. Within 48 hours, she was unable to walk and developed urinary urgency progressing to retention and constipation.

**Figure 1:**
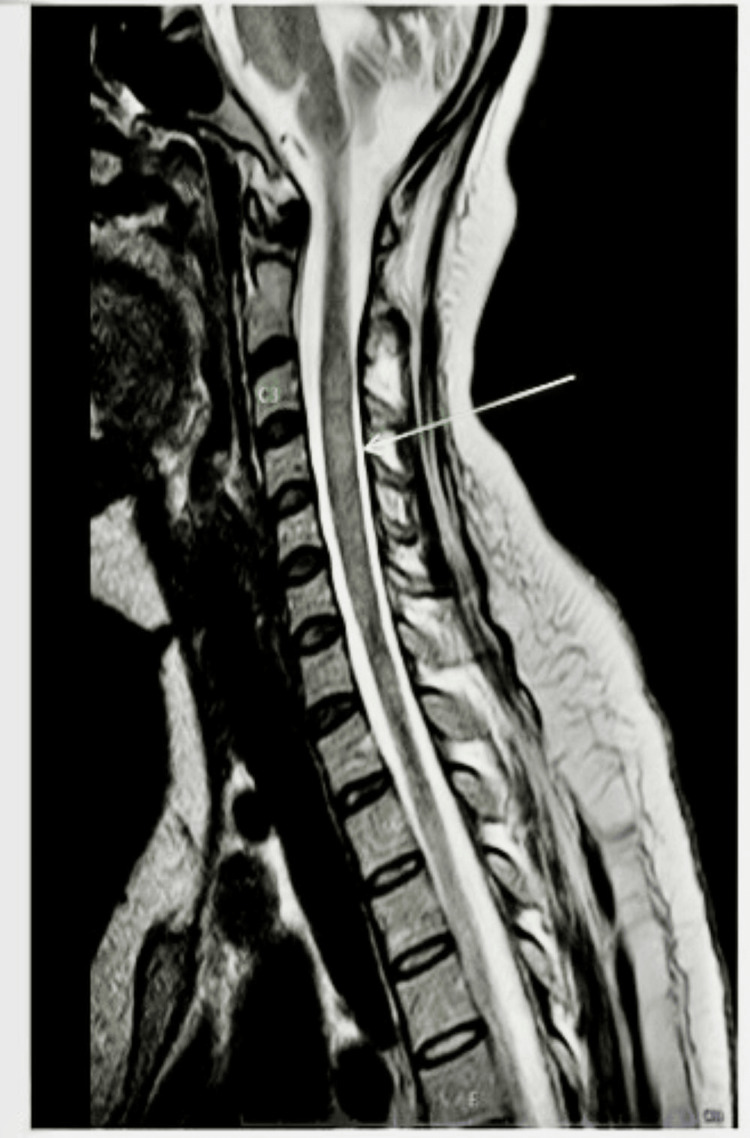
MRI spine (T2-weighted sagittal section) shows a hyperintense longitudinally extensive lesion extending from C3 to T2 Arrow indicates the area of spinal cord involvement

**Figure 2:**
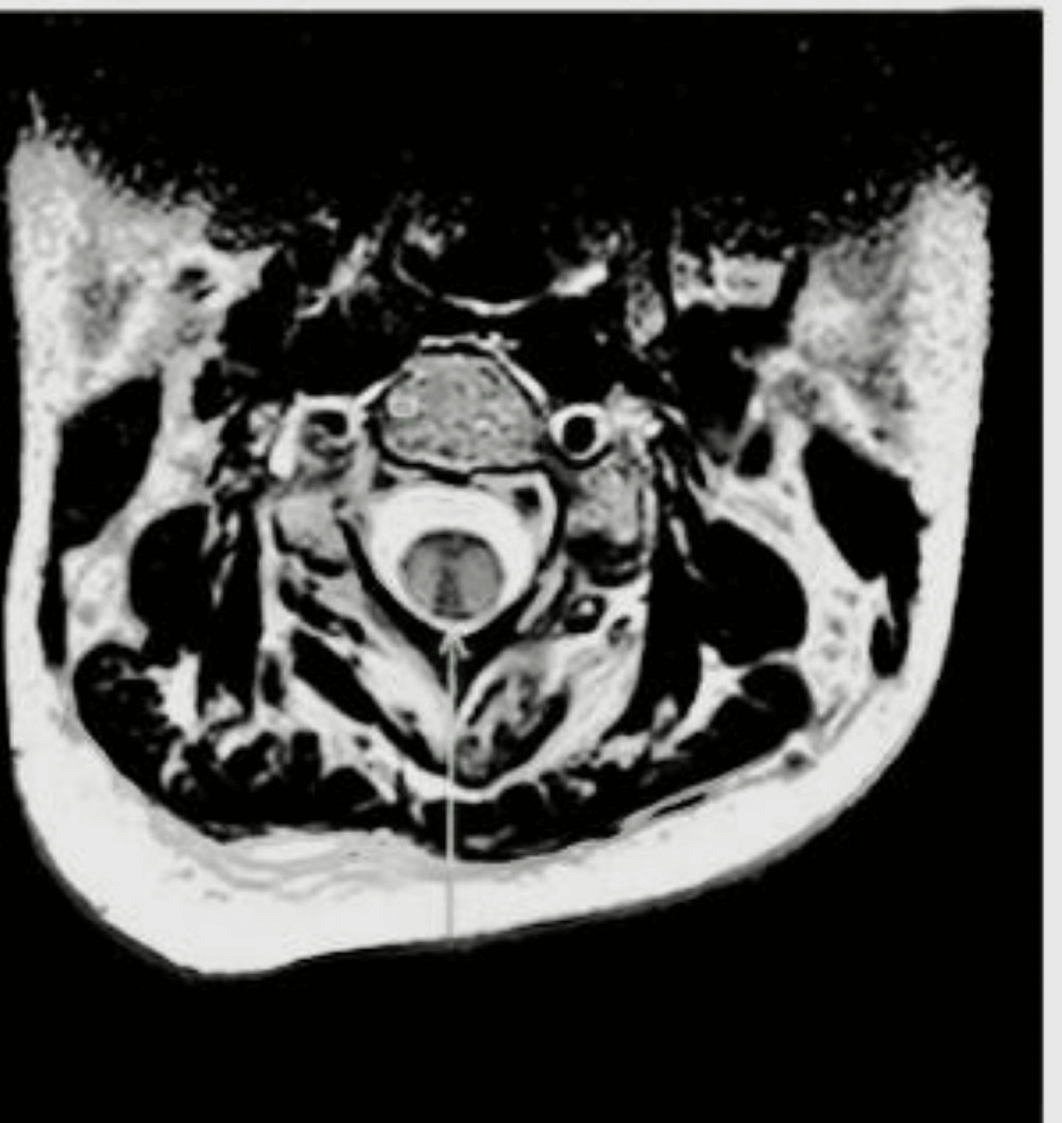
MRI spine (T2-weighted axial section) demonstrates the hyperintensity in the cervical spine Arrow highlights the hyperintensity

Neurological examination revealed normal cranial nerves, upper limb power of 4/5, and lower limb power of 1/5 with brisk reflexes, with reduced pain, temperature sensation till the T4 sensory level. MRI spine revealed longitudinally extensive T2-hyperintense lesions from C3 to T2 (Figures 1, 2).

Infectious and autoimmune panels were negative, including ANA, ANCA, serum anti-AQP4, and anti-myelin oligodendrocyte glycoprotein (anti-MOG) antibodies. Blood and urine cultures were unremarkable. Broad laboratory workup evaluating for causes of transverse myelitis was also found to be negative (Table 1).

**Table 1:** Laboratory serum values SSA: Sjogren syndrome-related antigen A autoantibodies; SSB: Sjogren syndrome-related antigen B autoantibodies; IgG: immunoglobulin G; NMO: neuromyelitis optica, AQP4: anti-aquaporin 4 antibody; MOG: myelin oligodendrocyte glycoprotein; HbA1C: glycated hemoglobin, HIV: human immunodeficiency virus

Parameters	Normal value	Patient value
SSA (Ro) antibody (U/L)	<5	2
SSB (La) antibody (U/L)	<5	0
Antinuclear antibody	Negative	Negative
Rheumatoid factor (U/L)	<15	<15
NMO/AQP4	Negative	Negative
MOG	Negative	Negative
Copper (mcg/dL)	62-140	120
Ceruloplasmin level (mg/dL)	20-40	30
Vitamin B12 level (pg/mL)	190-950	350
HbA1c (%)	<5.7	5.1
Rapid plasma reagin	Non-reactive	Non-reactive
HIV Ab	Non-reactive	Non-reactive

She underwent a lumbar puncture, and cerebrospinal fluid analysis showed 450 cells/mm³, predominantly lymphocytes (lymphocytic pleocytosis)with normal glucose and protein (Table 2). Cerebrospinal fluid (CSF) anti-neuromyelitis optica (anti-NMO) and anti-MOG were negative. She had mild leukocytosis (total leucocyte count of 14,000) with mildly raised C-reactive protein (CRP; 12.28).

**Table 2:** Cerebrospinal fluid (CSF) analysis WBC: white blood cell; RBC: red blood cell; IgG: immunoglobulin G; ACE: angiotensin-converting enzyme; PCR: polymerase chain reaction; CMV: cytomegalovirus, VZV: varicella-zoster virus; HSV: herpes simplex virus, HHV: human herpes virus

Parameters	Normal value	Patient value
WBC (cells/mcL)	0-5	450 cells lymphocyte predominant
RBC (cells/mcL)	0-2	700
Glucose (mg/dl)	40-70	67
Protein (mg/dl)	15-45	155
Oligoclonal bands	0	0
IgG index (U)	0.32 -0.60	0.5
ACE level (U/L)	0-2.5	1.5
CSF PCR	Undetectable for Cytomegalovirus, Varicella zoster virus, HSV-1, HSV-2, HHV-6, Enterovirus, Cryptococcus, Haemophilus influenzae, Listeria monocytogenes, Neisseria meningitidis, Streptococcus agalactiae, and Streptococcus pneumoniae	
CSF Gene Xpert	Negative	Negative
CSF culture	Negative	Negative
CSF fungal culture	Negative	Negative

She received intravenous methylprednisolone (1 gram per day for five days) and intravenous immunoglobulin (2 grams per kg over five days) along with an antibiotic, ceftriaxone. Her upper limb strength improved to 5/5 and lower limb to 3/5 within two weeks. At 35 weeks, she developed a urinary tract infection with sepsis due to a sensitive strain of *Klebsiella pneumoniae* and underwent emergency cesarean section under general anesthesia. She delivered a preterm infant (1.84 kg, APGAR score 4/10) requiring neonatal intensive care unit (NICU) admission.

Postpartum, she improved with physiotherapy and neurorehabilitation, regaining full lower limb strength by two months and residual mild bladder urgency.

## Discussion

Transverse myelitis exhibits a bimodal age distribution, commonly affecting individuals in their second and fourth decades. In contrast to this occurrence, our patient was affected in her third decade of life [4]. It is extremely rare during pregnancy. During the acute phase of illness in NMOSD, AQP4 IgG antibodies produced from the hypothalamus cross the placenta and bind with AQP4 on placental syncytiotrophoblasts. However, the expression of AQP4 channels is reduced from the first to the third trimester [5].

Our patient was diagnosed with acute transverse myelitis in the third trimester, and hence, the decreased load of autoantibodies crossing the placenta. However, an acute transverse myelitis (ATM) can be life-threatening, leading to complications such as premature delivery. Our patient had sepsis due to a urinary tract infection at 35 weeks of gestation. Therefore, she underwent an emergency cesarean section and delivered a premature baby. It can result from diverse etiologies, including infectious, autoimmune, demyelinating, paraneoplastic, and drug-induced causes; however, many remain idiopathic [6,7].

Despite laboratory evidence of the causative organism, the presence of contrast-enhanced MRI with lymphocytic pleocytosis in CSF was helpful to narrow down the diagnosis of para-infectious transverse myelitis. It varies, with only one-third of patients achieving complete recovery [7]. First-line therapy for TM includes high-dose intravenous corticosteroids, followed by plasma exchange in steroid-refractory cases [7]. Intravenous immunoglobulin (IVIG) has been reported as a useful alternative, particularly when plasmapheresis poses risks during pregnancy.

Plasmapheresis carries maternal risks such as hemodynamic instability, electrolyte imbalance, and coagulopathy, and fetal complications like distress and preterm contractions [8]. In our patient, IVIG use was effective and well-tolerated, resulting in near-complete neurological recovery, suggesting IV immunoglobulin as a alternative promising option in the management of acute transverse myelitis in pregnancy.

## Conclusions

This case report highlights the rare occurrence of parainfectious transverse myelitis during pregnancy. Early diagnosis and timely initiation of immunomodulatory therapy, particularly high-dose corticosteroids and intravenous immunoglobulin, can lead to substantial neurological improvement and favorable obstetric outcomes. Our patient demonstrated remarkable, favorable, significant neurological recovery with timely therapy. This report further reinforces the need for multidisciplinary care involving neurology, obstetrics, and critical care teams to ensure optimal maternal and fetal safety. Overall, this case adds to the limited literature on pregnancy-associated parainfectious myelitis and emphasizes that early recognition and aggressive treatment remain key determinants of prognosis.
